# The phylogeny and metabolic potentials of an *n*-alkane-degrading *Venatorbacter* bacterium isolated from deep-sea sediment of the Mariana Trench

**DOI:** 10.3389/fmicb.2023.1108651

**Published:** 2023-03-22

**Authors:** Jiahua Wang, Yan Zhang, Ying Liu, Zhe Xie, Junwei Cao, Hongcai Zhang, Jie Liu, Tianqiang Bao, Congwen Sun, Bilin Liu, Yuli Wei, Jiasong Fang

**Affiliations:** ^1^Shanghai Engineering Research Center of Hadal Science and Technology, College of Marine Sciences, Shanghai Ocean University, Shanghai, China; ^2^State Key Laboratory of Marine Geology, Tongji University, Shanghai, China; ^3^Laboratory for Marine Biology and Biotechnology, Qingdao National Laboratory for Marine Science and Technology, Qingdao, China

**Keywords:** Trench sediment, *Venatorbacter*, n-alkane utilization, environmental distribution, extracellular macromolecule degradation

## Abstract

Recently, several reports showed that *n-*alkanes were abundant in the hadal zone, suggesting that *n-*alkanes could be an important source of nutrients for microorganisms in hadal ecosystems. To date, most of the published studies on the microbial capacity to degrade hydrocarbons were conducted only at atmospheric temperature and pressure (0.1 MPa), and little is known about whether and which microbes could utilize *n*-alkanes at *in situ* environmental conditions in the hadal zone, including low temperature and high hydrostatic pressure (especially >30 MPa). In this study, a piezotolerant bacterium, strain C2-1, was isolated from a Mariana Trench sediment at depth of 5,800 m. Strain C2-1 was able to grow at *in situ* temperature (4°C) and pressure (58 MPa) with *n-*alkanes as the sole carbon source. Phylogenetically, strain C2-1 and related strains (TMPB967, ST750PaO-4, IMCC1826, and TTBP476) should be classified into the genus *Venatorbacter*. Metagenomic analysis using ~5,000 publicly available datasets showed that *Venatorbacter* has a wide environmental distribution in seawater (38), marine sediments (3), hydrothermal vent plumes (2), Antarctic ice (1), groundwater (13), and marine sponge ecosystems (1). Most *Venatorbacter* species are non-obligate *n-*alkane degraders that could utilize, at a minimal, C_16−_C_18_ *n*-alkanes, as well as other different types of carbon substrates, including carbohydrates, amino acids, peptides, and phospholipids. The type II secretion system, extracellular proteases, phospholipase, and endonuclease of *Venatorbacter* species were robustly expressed in the metatranscriptomes of deep-sea hydrothermal vents, suggesting their important contribution to secondary productivity by degrading extracellular macromolecules. The identification of denitrifying genes suggested a genus-specific ecological potential that allowed *Venatorbacter* species to be active in anoxic environments, e.g., the oxygen-minimal zone (OMZ) and the deeply buried marine sediments. Our results show that *Venatorbacter* species are responsible for the degradation of hydrocarbon and extracellular macromolecules, suggesting that they may play an important role in the biogeochemistry process in the Trench ecosystems.

## 1. Introduction

*n*-Alkanes are aliphatic hydrocarbons derived from biological and/or thermogenic sources (Rojo, 2009). It has been estimated that marine algae produce 308–771 million tons of predominantly mid-length, straight-chain aliphatic hydrocarbons (primarily C_15_ and C_17_ alkanes) annually (Schirmer et al., 2010; Lea-Smith et al., 2015; Valentine and Reddy, 2015). The estimated production of biogenic alkanes in marine systems far exceeds the input from oil spills (Lea-Smith et al., 2015; Love et al., 2021) and natural seeps (White et al., 2019), which could sustain the growth of hydrocarbon-degrading microbes, including *Alcanivorax, Alteromonas, Cycloclasticus, Bacillus, Flavobacterium, Marinobacter, Pseudomonas, Thalassolituus*, and *Rhodococcus* (Harayama et al., 2004; Yakimov et al., 2007; Rojo, 2009).

Many environmental factors may impact the microbial degradation of *n-*alkanes, including temperature and hydrostatic pressure (Schedler et al., 2014; Perez Calderon et al., 2019). It was reported that low temperature limits the growth of *n-*alkane degraders. For example, *Alcanivorax* species could not grow below 10°C, although they were isolated from 2,682 to 5,000 m in the deep sea (Liu and Shao, 2005; Lai et al., 2011, 2013). High hydrostatic pressure also affects *n-*alkane degradation, and a modest increase in hydrostatic pressure from 0.1 to 5 MPa was sufficient to affect cell replication in *Alcanivorax dieselolei* KS_293 and *Alcanivorax jadensis* KS_339 (Scoma et al., 2016). Further increase to 10 MPa decreased the growth of *A. dieselolei* KS_293, in addition to a general downregulation of genes for *n-*alkane degradation (Scoma et al., 2016). For type strain *A. borkumensis* SK2^T^, the increased cell damage at 10 MPa was consistent with the intracellular accumulation of piezolyte ectoine (Scoma and Boon, 2016). Similarly, the growth of the aromatic hydrocarbon-degrading strain *Sphingobium yanoikuyae* B1^T^ was highly affected by elevated pressures that 12 MPa or higher pressure abolished its growth (Schedler et al., 2014).

The hadal zone (6,000–11,000 m) is the deepest part of the ocean on earth. The hadal zone is featured with extreme conditions, including darkness, high hydrostatic pressure (HHP, up to ~110 MPa), low temperature (1.0–2.5°C), low dissolved oxygen (~156 μM), and high heterogeneity of organic matter (Jamieson et al., 2010; Cressey, 2015; Liu et al., 2018). The nitrate concentration in the deep ocean was estimated to be 16–43 μmol/kg, which was higher than those in the surface waters (0–30 μmol/kg) (Nagata et al., 2010) and could be a complementary electron acceptor of the microbes living in the hadal zone with low dissolved oxygen. Many deep marine ecosystems, such as the sediments of cold seeps and hydrothermal vents, have been reported with replete hydrocarbons (Kallmeyer and Boetius, 2004; Orcutt et al., 2010; Musat, 2015). Recently, Guan et al. (2019) also reported that the concentrations of *n-*alkanes ranged from 0.34 to 3.47 μg/g dry weight (dw) at 4,900–7,068 m in Mariana Trench sediments (Guan et al., 2019). Liu et al. (2019) reported an average concentration of 2.3 μg/g dw *n*-alkane at the bottom of Mariana Trench (Liu et al., 2019). Some hydrocarbonoclastic microbes from the hadal biospheres have been isolated, e.g., *A. jadensis* ZYF844, *Alcanivorax venustensis* ZYF848, and *A. dieselolei* ZYF854 from the seawater of the Mariana Trench at 10,400 m (Liu et al., 2019) and *Thalassolituus* *alkanivorans* TTPB476 from the water column of the Mariana Trench at 6,000 m (Wei et al., 2022). In addition, metagenomic analysis suggested that some hadal microbes could potentially utilize *n-*alkanes (Liu et al., 2019). These studies suggest that *n*-alkanes may be an important source of nutrients in hadal ecosystems. However, most of these studies on microbial degradation of *n*-alkanes were conducted at atmospheric temperature and pressure, and little is known about which microbes could function as active *n-*alkane degraders at the low-temperature and HHP conditions of the hadal zones.

In this study, a gammaproteobacterial strain C2-1 was isolated from Mariana Trench sediments. The *n-*alkane-degrading capability of strain C2-1 was confirmed in laboratory simulation under both *in situ* and atmospheric pressures and temperatures. The phylogeny, environmental distribution, and metabolic potentials of the C2-1-like strains were further studied with ~5,000 publicly available metagenomic and metatranscriptomic datasets. Our study revealed for the first time the accurate phylogeny, extensive environmental distributions, and diverse metabolic potentials of *Venatorbacter* species in the shallow and deep marine environments and highlighted its unique role in the biogeochemical cycle of the deep ocean.

## 2. Materials and methods

### 2.1. Sample description

Sediment samples were collected at depth of 5,800 m (142.274°E, 10.761°N) in the Mariana Trench during the cruise aboard R/V “ZHANG JIAN HAO” in December 2017. A box corer (with a base area of 400 cm^2^ and a height of 25 cm) attached to the Hadal Lander II was used to collect sediment samples from the Trench as described in Luo et al. (2018). To collect sediment from the seafloor, 2 h after the lander reached the seafloor, the box corer was slowly driven into the seafloor until it reached ~25 cm below the sediment surface. A lid was then released to seal the box corer, and the lander was recovered. After recovering on board, the sediment core was immediately subsampled and stored in 50-ml sterile centrifuge tubes in a cold room at 4°C on board the ship.

### 2.2. Bacterial enrichment and isolation

Approximately 2.5 g of the collected sediment from the middle of the core was used to set up enrichment cultures for bacterial isolation at each pressure (10, 20, 50, and 100 MPa). The sediment was added into 100 ml MMC medium (24 g/L NaCl, 7.0 g/L MgSO_4_·7H_2_O, 1 g/L NH_4_NO_3_, 0.7 g/L KCl, 2.0 g/L KH_2_PO_4_, 3.0 g/L Na_2_HPO_4_, and pH 7.5) (Liu et al., 2019), supplemented with 0.1 ml of a vitamin mixture (including 40 mg/L 4-aminobenzoic acid, 10 mg/L biotin, 100 mg/L nicotinic acid, 50 mg/L pantothenic calcium, 150 mg/L pyridoxine dihydrochloride, 100 mg/L thiamine chloride dihydrochloride, and 50 mg/L cyanocobalamin), 0.1 ml of a trace element mixture [including 10 mg/L H_3_BO_3_, 5 mg/L MnCl_2_·4H_2_O, 4 g/L FeSO_4_·7H_2_O, 190 mg/L CoCl_2_·6H_2_O, 24 mg/L NiCl_2_·6H_2_O, 2 mg/L CuCl_2_·2H_2_O, 200 mg/L ZnSO_4_·7H_2_O, 36 mg/L Na_2_MoO_4_·2H_2_O, and 13 ml/L HCl (7.7 M)], and 125 mg/L *n-*alkane mixture (hexadecane, heptadecane, and octadecane as 1:1:1, w/w) as the carbon sources, and filled into disposable plastic syringes, which were sterilized by ultraviolet light before usage. After heat sealing, these disposable plastic syringes were placed into stainless steel high-pressure vessels (Feiyu Science and Technology Exploitation Co. Ltd., Nantong, China) and incubated at 4 °C and multiple pressures (10, 20, 50, and 100 MPa).

After 6 months of incubation, the enrichment cultures were diluted (1:1,000) and then coated in MMC-alkane-agar plates at 4°C and atmospheric pressure for bacterial isolation. After another 6 months, bacterial colonies, presenting no more than 10 per plate, were picked, inoculated on R2A-agar plates (containing 0.5 g/L tryptone, 0.5 g/L yeast extract, 0.5 g/L soluble starch, 0.5 g/L glucose, 0.5 g/L casein amino acids, 0.3 g/L sodium pyruvate, and 15 g/L agar power, pH = 7.2), and then cultured at 16°C. Such adjustments in temperature, pressure, and culture medium could accelerate its growth for better identification of its colony morphology. After 1 week, strain C2-1 (= MCCC 1K03735 = KCTC 72289) was isolated from the R2A-agar plates.

### 2.3. Chemotaxonomy

For cellular fatty acid analysis, strain C2-1 and its closely related reference strains (*Thalassolituus marinus* IMCC1826^T^, *Thalassolituus oleivorans* DSM 14913^T^, *Oleibacter marinus* DSM 24913^T^, and *Oceanobacter kriegii* IFO 15467^T^) were cultured in MB for 3 days at 35°C. Fatty acids were saponified, methylated, and extracted using the standard protocol of MIDI (Sherlock Microbial Identification System, version 6.0). Fatty acid methyl esters were analyzed by gas chromatography (Agilent Technologies 6850) and identified by using the RTSBA6.0 database of the Microbial Identification System (Athalye et al., 1985). Polar lipids of strain C2-1^T^ were extracted and separated on silica gel 60 F_254_ aluminum-backed thin-layer chromatography plates (10 × 10 cm; Merk 5554) and further analyzed according to Minnikin et al. (1984). The first dimension of the solvent system was chloroform/methanol/water (65:24:4, by vol.), and the second dimension was chloroform/glacial acetic acid/methanol/water (80:15:12:4, by vol.). Then, the plates were sprayed with 5% phosphomolybdic acid (w/v, dissolved in alcohol) and heated at 160°C for 10–15 min to reveal total lipids. The respiratory quinones were extracted using the method described by Minnikin et al. (1984) and analyzed by HPLC as described by Tindall (1990).

### 2.4. Microbial degradation of *n*-alkanes

The MMC liquid media (30 ml), prepared as described earlier, was distributed in air-tight syringes and inoculated with freshly grown strain C2-1. The cultures were supplemented with a mixture of C_16 − 18_ *n*-alkanes as the sole source of carbon, and the culture medium was then filled into disposable plastic syringes, which were sterilized by ultraviolet light before usage. After heat sealing, these disposable plastic syringes were placed into stainless steel high-pressure vessels (Feiyu Science and Technology Exploitation Co. Ltd., Nantong, China). High-pressure incubations were conducted at 20°C and atmospheric pressure (0.1 MPa) or at *in situ* temperature and pressure (4°C and 58 MPa) for 10 days.

Once the incubation was terminated, the cultures were extracted for *n*-alkanes using dichloromethane (DCM) twice. The extracts were combined, dried under a gentle stream of nitrogen, and then redissolved in 500 μl DCM. The extracted *n-*alkanes were analyzed on a Hewlett-Packard 7890B gas chromatography with an HP 5977A Mass Selective Detector. Analytical separation of the *n*-alkanes was accomplished using a HP-5ms capillary column (50 m × 0.25 mm × 0.25 μm). The column temperature was programmed from 70 to 130°C at 20°C/min and then to 310°C at 4°C/min (and held for 15 min). Helium was used as a carrier gas, and the flow rate was set at 1 ml/min. The concentrations of individual *n*-alkanes were determined based on the chromatographic response of the alkanes relative to that of the internal standard (squalene, Sigma).

### 2.5. Genomic DNA extraction, sequencing, and assembly

The genomic DNA of strain C2-1 was extracted as described by Fang et al. (2017). The genome of C2-1 was sequenced using a combination of PacBio RS II Single Molecule Real Time (SMRT) and Illumina HiSeq 2500 platforms by MajorBio (Shanghai Co., Ltd., China).

For Illumina sequencing, approximately 1μg genomic DNA was sheared into 400–500 bp fragments using a Covaris M220 Focused Acoustic Shearer following the manufacturer's protocol. Illumina sequencing libraries were prepared from the sheared fragments using the NEXTFLEX™ Rapid DNA-Seq Kit.

For Pacific Biosciences sequencing, an aliquot of 15 μg DNA was spun in a Covaris g-TUBE (Covaris, MA) at 6,000 RPM for 60 s using an Eppendorf 5424 centrifuge (Eppendorf, NY). DNA fragments were then purified, end-repaired, and ligated with SMRTbell sequencing adapters following the manufacturer's recommendations (Pacific Biosciences, CA). The resulting sequencing library was purified three times using 0.45 × volumes of Agencourt AMPure XP beads (Beckman Coulter Genomics, MA) following the manufacturer's recommendations. Next, a ~10 kb insert library was prepared and sequenced on one SMRT cell using standard methods.

The data generated from PacBio and Illumina platforms were used for bioinformatics analysis. The complete genome sequence was assembled using both the PacBio reads and the Illumina reads. The original image data are transferred into sequence data *via* base calling, which is defined as raw data or raw reads and saved as a FASTQ file. A statistic of quality information was applied for quality trimming, by which the low-quality data can be removed to form clean data. The reads were then assembled into a contig using the hierarchical genome assembly process (HGAP) and canu (Koren et al., 2017). The last circular step was checked and finished manually, generating a complete genome with a single seamless chromosome. Finally, error correction of the PacBio assembly results was performed using the Illumina reads using Pilon (Walker et al., 2014).

### 2.6. Gene annotation

The NCBI prokaryotic genome annotation pipeline (Tatusova et al., 2016) was used in ORF prediction and gene annotation. The predicted protein sequences were also aligned with the Clusters of Orthologous Groups of proteins (COG) (Galperin et al., 2021) and TransporterDB 2.0 (Elbourne et al., 2017) databases using the BLASTp software with the following parameters: identity, 50%; query-cover, 80%; and *e*-value 1e-5. The annotation of the Kyoto Encyclopedia of Genes and Genomes (KEGG) was assigned with BlastKOALA (Kanehisa et al., 2016). The genomic islands were predicted using IslandViewer 4 (Bertelli et al., 2017).

### 2.7. Phylogenetic analysis

The 120 conserved bacterial marker genes of the GTDB taxonomy were used to study the phylogeny of strain C2-1 and its related strains. The sequences of 120 concentrated proteins in the genomes were predicted using GTDB-Tk (database version: Release 07-RS207) (Chaumeil et al., 2019) and separately aligned using Clustal Omega (Sievers and Higgins, 2021). The aligned sequences were manually degapped. If one concentrated protein was not identified in some genome, we would then add the corresponding number of “–,” depending on the length of the sequence after degap. Then, the degapped alignments of each concentrated protein were tandemly connected (Tang et al., 2021). The phylogenetic tree was constructed using FastTree2 with the neighbor-joining method (Price et al., 2010), and a bootstrap analysis with 1,000 replicates was performed to assess the robustness of the tree. Finally, the phylogenetic tree was plotted using iTOL (Letunic and Bork, 2021).

In addition, to identify the genus-specific insertions and deletions of genus “UBA2009,” we separately aligned the 120 conserved proteins predicted by GTDB-tk (Chaumeil et al., 2019) using Clustal Omega (Sievers and Higgins, 2021) and then identified the insertions and deletions based on the alignments using lab-in Perl scripts.

### 2.8. Search and retrieval of *Venatorbacter* genomes from large-scale metagenomic datasets

To study the geographic distribution of the genus *Venatorbacter*, we first identified three signature proteins of this genus (FOT50_RS00155, FOT50_RS08930, and FOT50_RS12870 in C2-1), which were annotated as hypothetical proteins, and found in almost all the *Venatorbacter* genomes, but absent in any other microbes (BLASTp identity, 50%; query-cover, 80%). Then, these signature proteins were used to query our in-house database which includes the protein sequences from 4,830 publicly available environmental metagenomic datasets (human-associated samples were excluded). If one sample contained at least two signature proteins, the reads would be trimmed by Sickle v1.33 (https://github.com/najoshi/sickle) and assembled using MEGAHIT (Li et al., 2016) with the following parameters: min kmer 31; max kmer, 149; and kmer step 6. The reads were aligned to the contigs using Bowtie 2 (Langmead and Salzberg, 2012). A BamM (v.17.3, https://github.com/ecogenomics/BamM) filter was used to screen the reads mapped to the contigs with coverage ≥90% and identity ≥95%. The average per-base-pair coverage of the contigs was calculated by BamM “parse” with the parameter “tpmean” to remove the top and bottom 10% coverage regions. The contigs longer than 2.5 kb were used to calculate contig coverages and perform binning.

The protein sequences were predicted from the contigs using Prodigal (Hyatt et al., 2010) and then aligned with the signature proteins. The t-SNE algorithm-based dimension reduction from the tetranucleotide frequency matrix and visualization were performed using the R package mmgenome2 (https://github.com/KasperSkytte/mmgenome2). The signature protein-containing contigs were highlighted when visualization and manual binning were adopted to carefully divide the boundaries between metagenome-assembled genomes (MAGs). Rebinning was performed by plotting all the contigs from each bin based on their abundances plus GC content, and contigs with inconsistent coverage were manually deleted. The phylogeny of the MAGs was checked by GTDB-tk (Chaumeil et al., 2019), and the MAGs assigned as “UBA2009” were retained. Completeness and contamination of the MAGs were assessed using CheckM (Parks et al., 2015), and the MAGs with both >30% completeness and <10% contamination were finally retained.

### 2.9. Metatranscriptomic analysis

The clean datasets of SAMN03609707 and SAMN03609696 were downloaded from the NCBI SRA database and trimmed from raw reads with Trimmomatic (Bolger et al., 2014) for further analysis by removing adapter sequences, low-quality reads (*Q* value <20), ambiguous “N” nucleotides, and fragments of <35 bp. Then, the ribosomal reads were identified and removed using SortMeRNA (Kopylova et al., 2012). The non-ribosomal reads were mapped to the coding regions of their corresponding MAGs (SRR2046235_bin1 and SRR2046236_bin1) using Bowtie 2 (Langmead and Salzberg, 2012). The coverages of the genes were calculated by the same methods in metagenomic analysis.

## 3. Results

### 3.1. Description of strain C2-1

A small colony C2-1 was picked from the MMC-alkane-agar plates and then inoculated on R2A-agar plates, which formed clear, round colonies with regular, slightly raised edges. The transmission electron micrograph (TEM) showed that the cell of strain C2-1 was 1.15–1.52 μm in length, 0.19–0.37 μm in width, and 1.43–2.78 μm in flagellar length (Supplementary Figure S1). Cultured in the R2A medium, the optimum growth pressure for strain C2-1 was 0.1 MPa, with tolerance up to 50 MPa, indicating that C2-1 was a piezotolerant strain (Supplementary Figure S2).

The major cellular fatty acids of strain C2-1 (>5.0%) included Summed Feature 3 (C_16:1_ω7c and/or C_11:1_ω6c; 50.9%), C_16:0_ (23.8%), and Summed Feature 8 (C_18:1_ω7c and/or C_18:1_ω6c; 8.0%; Supplementary Table S1). The polar lipids of strain C2-1 were composed of phosphatidylglycerol (PG) and phosphatidylethanolamine (PE) (Supplementary Figure S3, Supplementary Table S1). The respiratory quinone of strain C2-1 was identified as ubiquinone Q-9.

### 3.2. The phylogenetic characteristics of strain C2-1

The alignments of 16S rRNA gene sequences showed that strain C2-1 is closely related to *Thalassolituus* *alkanivorans* TMPB967, *Thalassolituus* *marinus* IMCC1826, and *Venatorbacter cucullus* ASxL5^T^, with identities of 100, 96.98, and 96.29, respectively (Supplementary Table S2). However, the 16S rRNA similarity between TMPB967 and the *Thalassolituus* type strain *T. oleivorans* MIL-1^T^ was only 95.74%, suggesting that the genus *Thalassolituus* might be non-monophyletic.

**Figure 1:**
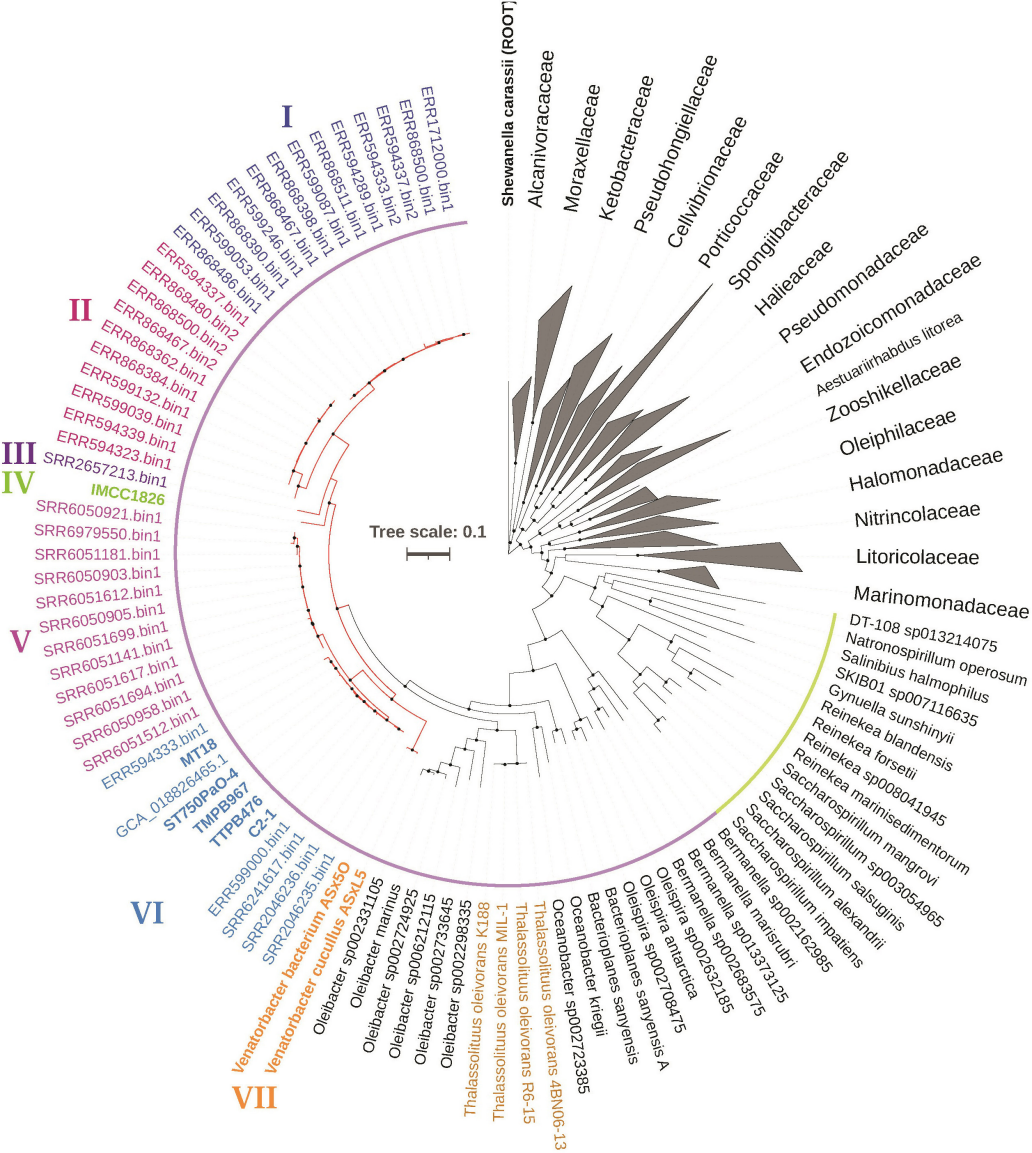
Phylogeny of *Venatorbacter* species and their related species based on 120 concentrated proteins. The two branches of the family Saccharospirillaceae in GTDB taxonomy were highlighted in yellow and purple, respectively. The branches of *Venatorbacter* are highlighted in red. Clades I to VII of *Venatorbacter* are in different colors. The genus *Thalassolituus* is highlighted in brown. The black nodes point to the bootstrap values ≥80.

Considering that *Venatorbacter cucullus* ASxL5^T^ was reported with heterogeneous 16S rRNA sequences, we obtained the complete genome sequences of strain C2-1 (Table 1, Supplementary Figure S4) and constructed a phylogenetic tree based on 120 conserved protein sequences (known as GTDB taxonomy). It showed that strain C2-1, TMPB967, IMCC1826, TTBP476, and 41 metagenome-assembled genomes (see below) were classified as “UBA2009” in GTDB taxonomy and formed a branch together with *Venatorbacter cucullus* ASxL5^T^, a recently reported *Oceanospirillales* predator isolated from a bovine slurry tank. This branch was divided from the *Thalassolituus* strains, *T. oleivorans* MIL-1^T^, R6-15, 4BN06-13, and K188 (Figure 1).

**Table 1:** Genome features of strain C2-1.

**Items**	**Description**
Size (bp)	4,317,714
G + C content (%)	53.11
Coding sequence (%)	89.93
Total genes	3,905
Protein-coding genes	3,835
Genes assigned to COG	2,459
rRNA operons	4
tRNA genes	54
tmRNA genes	1
ncRNA genes	3
Pseudogene	26
Gene islands	13

Moreover, the GC contents of these “UBA2009” genomes (including MAGs) were 52.43%−56.12%, which are higher than those of *Thalassolituus* strains (45.52%−46.62%; Supplementary Table S3). In addition, some components of cellular fatty acids in strain C2-1 and IMCC1826 were also distinct from those of neighboring genera. For instance, C_12:0_ was one of the major fatty acids in *T. oleivorans* DSM 14913^T^, *O. marinus* DSM 24913^T^, and *O. kriegii* IFO 15467 (5.7%−12.1%) but was only 1.3% and 1.9% in strain C2-1 and IMCC1826, respectively, whereas C_12:1_ 3-OH was not detectable in other three strains but was up to 3.9 and 7.8% in strain C2-1 and IMCC1826, respectively (Supplementary Table S1). These pieces of evidence suggest that strain C2-1, TMPB967, ST750PaO-4, IMCC1826, and TTBP476 should be classified as members of *Venatorbacter*, and seven clades (Clade I-VII) of *Venatorbacter* were further proposed based on their phylogeny (Figure 1). The ANI and DDH values among the seven clades of *Venatorbacter* and *Thalassolituus* species are shown in Supplementary Tables S4, S5, respectively.

To further study the phylogenetic markers of the genus *Venatorbacter*, the 120 conserved protein sequences from *Venatorbacter* and the neighboring genera were aligned. One deletion with two amino acid residues in leucyl-tRNA synthetase (TIGR00396) was only identified in the genus *Venatorbacter* (Supplementary Figure S5). Moreover, three signature proteins (SPs) of *Venatorbacter* (FOT50_RS00155, FOT50_RS08930, and FOT50_RS12870 in C2-1) were identified and were found in all the *Venatorbacter* genomes but absent in any other microbes in the NCBI nr database. Such SPs as molecular markers could be used to search for *Venatorbacter* species from large-scale metagenomic datasets.

### 3.3. The environmental distribution of *Venatorbacter* species

To study the environmental distribution of bacteria in this novel genus, we employed the aforementioned signature proteins of *Venatorbacter* to search against the NCBI nr database and our in-house database, including the protein sequences predicted from ~5,000 metagenomic assemblies. As many as 41 MAGs of *Venatorbacter* were retrieved, which accounted for 0.1%−15.82% of the total reads (Supplementary Table S6). We also collected 16 16S rDNA amplicons which showed >97% identities with isolated *Venatorbacter* strains (Supplementary Table S6). In addition, the sampling information of strain MT-18 was also included, which we recently isolated from sediment at 11,000 m of the Mariana Trench (unpublished data).

**Figure 2:**
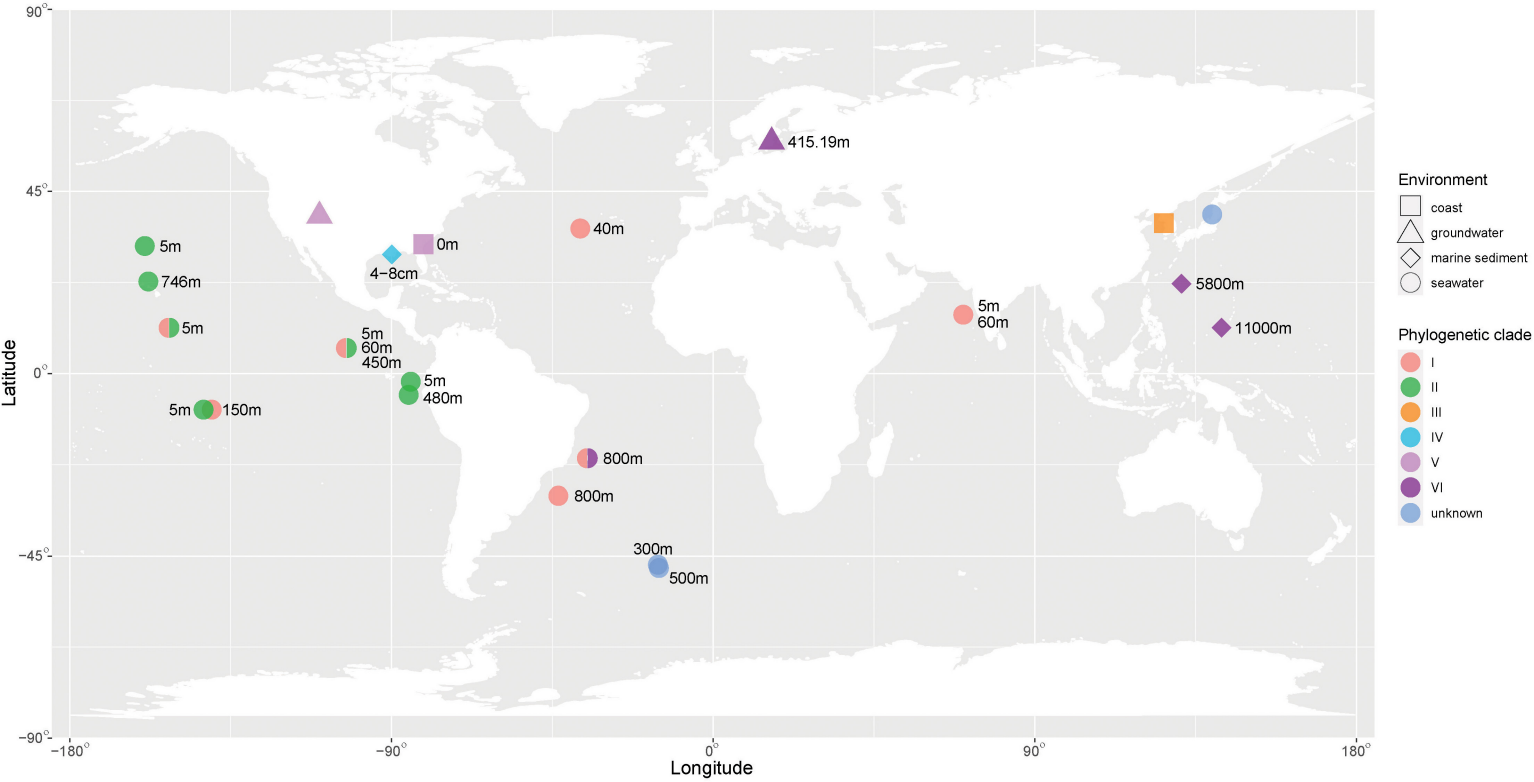
Environmental distribution of *Venatorbacter*. The phylogenetic clades of *Venatorbacter* are in different colors. The environmental information is shown in various shapes. The available depth of the sampling sites is also shown.

The environmental distribution of *Venatorbacter* is shown in Figure 2. It is clear that the genus *Venatorbacter* is widely distributed in various environments, including seawater (38 of sample locations), marine sediments (3), hydrothermal vent plumes (2), Antarctic ice (1), groundwater (13), and marine sponges (1) (Supplementary Table S6). Their distribution in the marine environments ranged from surface seawater to the deepest place on earth (11,000 m). Notably, the *Venatorbacter* MAGs were the most abundant microbes in samples from the Von Damm hydrothermal vent plume at a depth of 2,041 m (SAMN03609667) and 2,238 m (SAMN03609666) but were absent in the reference seawater sample from 2,337 m (SAMN03609668), indicating their important ecological function in the hydrothermal vent ecosystems, which will be further discussed below.

In addition, different *Venatorbacter* clades showed distinct distributions. Clades I and II were only observed in marine water less than 800 m in depth; Clades III (including strain IMCC1826^T^) and IV were only found in coastal waters and marine sediment, respectively, and Clade V was found in groundwater and coastal water. Interestingly, Clade VI (including C2-1, TMPB967, TTBP476, ST750PaO-4, MT-18, and six MAGs) was only observed in the marine water, sediments (>750 m), and groundwater from Aspo HRL located in the southeast of Sweden (Lat N 57 26 4 Lon E 16 39 36) at depth of 415.19 m (GCA_018826465.1), suggesting that strains in Clade VI could adapt well to and play an important role in the deep biosphere.

### 3.4. Metabolic capabilities of *Venatorbacter*

**Figure 3:**
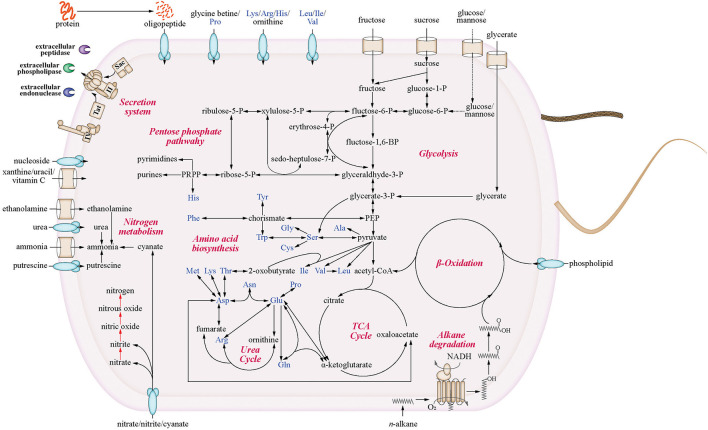
Metabolic potentials of strain C2-1 and IMCC1826^T^. Only the shared pathways of the two strains are shown. The red arrows highlight the genus-specific denitrification pathways that were identified in most genomes of this genus but absent in neighboring genera.

Given its wide distribution in the environment, we infer that *Venatorbacter* has diverse metabolic capabilities and possibly a wide range of environmental adaptation strategies. The reconstructed metabolic pathways of strains C2-1 and IMCC1826 showed the potential utilization of various carbon sources, including carbohydrates (fructose, sucrose, and glycerate), amino acids, oligopeptides, and phospholipids (Figure 3). In addition, the utilization of additional carbohydrates and organic acids was tested by Biolog GEN III and API 20NE, including glucose, mannose, xylitol, L-arabinose, D-arabitol, γ-amino butyric acid, and urocanic acid (Supplementary Table S7). In addition, the genes of type II secretion system (T2SS), preprotein translocation system, and many signal peptide-fused enzymes were identified in strains C2-1 and IMCC1826, suggesting that *Venatorbacter* were able to degrade extracellular components as carbon sources (see discussion).

**Figure 4:**
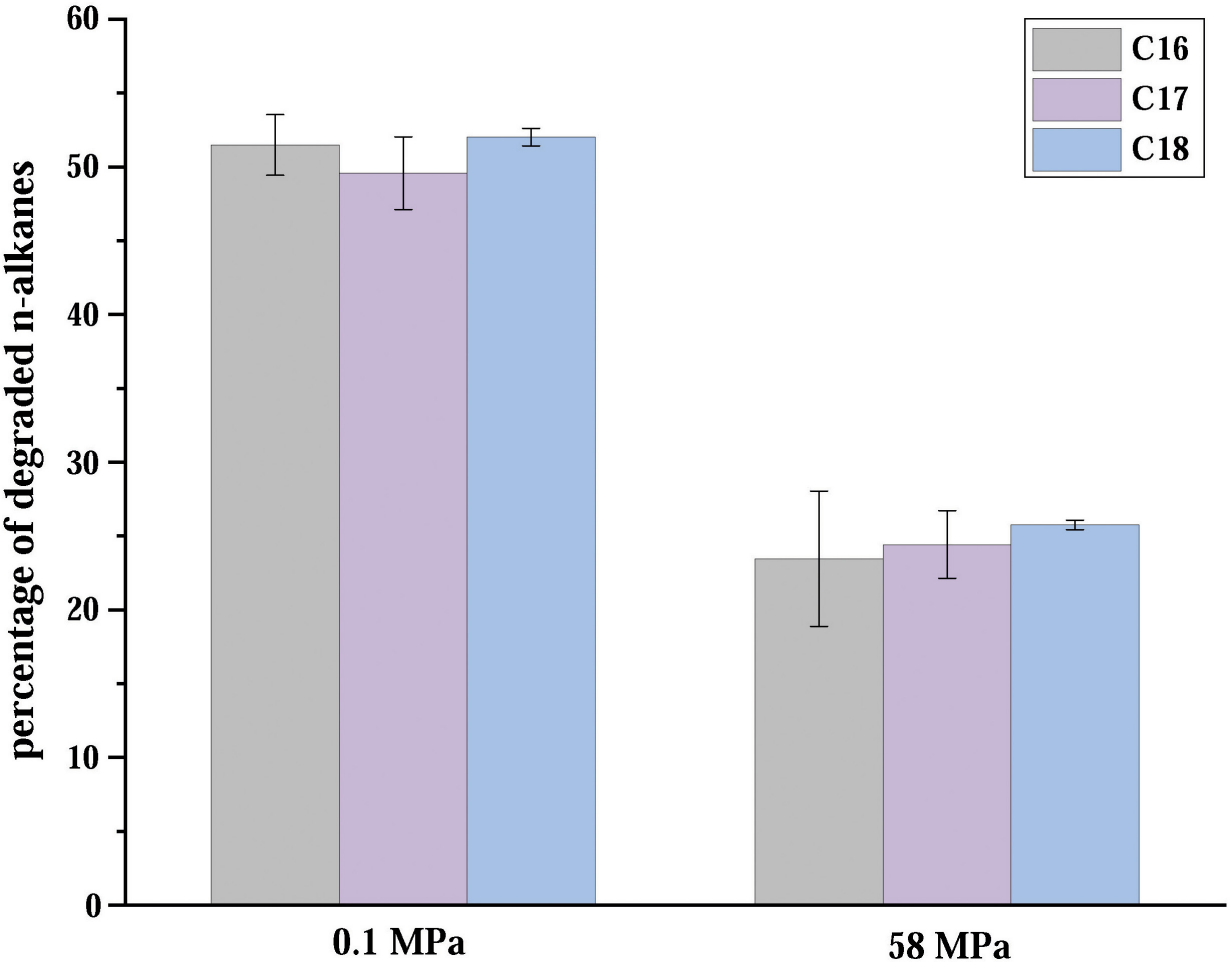
Percentages of C_16_-C_18_ straight-chain saturated *n-*alkanes degraded in 10 days in atmospheric and *in situ* conditions. The error bars are based on triplicate measurements.

Moreover, we found that C2-1 and most *Venatorbacter* genomes (including MAGs) retained two genes of non-heme diiron integral membrane *n-*alkane monooxygenases (*alkB*), *alkB*-dependent rubredoxin (*alkG*), and rubredoxin reductase (*alkT*) (Williams and Austin, 2022), suggesting that they could utilize *n*-alkane as carbon sources. Our results showed that ~50% of the *n-*alkanes were degraded at surface conditions (20°C and 0.1 MPa) after 10 days, and ~25% were degraded at *in situ* conditions (4°C and 58 MPa) when strain C2-1 was cultured with C_16_-C_18_ *n-*alkanes as the sole carbon source (Figure 4), indicating that *Venatorbacter* species could be *n*-alkane degraders under both surface water and deep-sea conditions.

For nitrogen metabolism, various transporters and metabolic pathways of nitrogen substrates were predicted in strain C2-1 and IMCC1826^T^, including ammonia, putrescine, urea, ethanolamine, methylamine, nitrate, nitrite, and cyanate (Supplementary Table S7). Interestingly, a complete pathway of denitrification was identified in almost all *Venatorbacter* genomes (including MAGs), comprising nitrate reductase (*narGHI*), nitrite reductase (*nirS*), nitric oxidase reductase (*norBC*), and nitrous oxide reductase (*nosZ*). Denitrification by strain C2-1 was further tested by our experiment (Supplementary Table S7), and similar results were obtained on IMCC1826^T^ in a previous study (Choi and Cho, 2013). Conversely, all the genomes from the neighboring genera (*Oleibacter, Thalassolituus, Oceanobacter, Bacterioplanes, Oleispira*, and *Bermanella*) were predicted to be deficient in denitrification. Considering that the expression of *narGHI* is typically induced at very low oxygen concentrations (Hoffmann et al., 2021), our results suggested a genus-specific ecological function of *Venatorbacter* in suboxic or anoxic environments, e.g., the oxygen-minimal zone (OMZ), where ST750PaO-4 was isolated from (BioSample: SAMN15097647), and the deeply buried marine sediments, where C2-1 and MT-18 were isolated from.

### 3.5. Adaptation to low temperature and high hydrostatic pressure

To reveal how *Venatorbacter* species in Clade VI adapt to the deep ocean environments, we studied the genes in C2-1 that were potentially involved in adaptation to stresses like high hydrostatic pressure (HHP) and low temperature (Supplementary Table S8). Previous studies have shown that betaine is one of the compatible solutes commonly found in microorganisms for osmoprotection, salt stress, and cold stress protection and has been found to be accumulated in deep-sea bacteria under HHP (Martin et al., 2002; Methe et al., 2005; Rath et al., 2020). In this study, we found that the genome of C2-1 contained genes encoding betaine aldehyde hydrogenase, choline dehydrogenase, choline transporting subunits, and a Bet I family regulator which regulates the biosynthesis of betaine from choline-containing compounds. A gene of the BCCT family transporter was also identified from its genome, which controls the input of betaine and other compatible solutes from the environment. The gene cluster (*ectABC*) encoding the enzymes for biosynthesis of ectoine (1,4,5,6-tetrahydro-2-methyl-4-pyrimidinecarboxylic acid) was detected in the genome of strain C2-1. Ectoine serves as a protectant in many bacterial cells against stresses such as high salinity, low temperature, and high temperature (Bursy et al., 2008; Graf et al., 2008; Ma et al., 2017). In addition, it plays an important role in stabilizing enzymes, DNA, and cytoplasmic membranes, which are prone to be affected at high pressure.

It has been shown that β-hydroxybutyrate (PHB) could protect deep-sea bacteria from high-pressure-induced protein structure changes (Martin et al., 2002). Although no biosynthetic gene of PHB was identified in C2-1, the genes encoding PHB depolymerase family esterase and D-(-)-3-hydroxybutyrate oligomer hydrolase were found, suggesting that strain C2-1 could potentially utilize PHB as an osmotic regulator in adaptation to variable osmotic environments by adjusting intercellular PHB molecular weight. In addition, strain C2-1 contained many chaperone genes including *hslR, dnaK, groEL*, and *cspD*. These chaperones were found to be induced at high pressure and/or low temperature, to help maintain protein folding (Simonato et al., 2006; Oger and Jebbar, 2010).

## 4. Discussion

It has been reported that abundant *n-*alkanes were accumulated in surface sediments of the Mariana Trench, which could be potentially from bacteria, algae, or contamination of diesel fuels (Guan et al., 2019; Liu et al., 2019). The high abundance of the potential hydrocarbon-degrading bacteria, together with the abundant *n*-alkane content in sediment, suggests that *n-*alkane-degrading microbes could contribute to carbon turnover in the sediment of the Mariana Trench (Guan et al., 2019). Although *n-*alkane degradation was observed in three *Alcanivorax* species isolated from near bottom water of the Mariana Trench at 60 MPa and 4°C (Liu et al., 2019), till now, no studies have shown that microorganisms isolated from the deep marine sediment are capable of *n-*alkane degradation at 4°C and exceeding 30 MPa. In this study, strain C2-1 can degrade aliphatic hydrocarbons, suggesting that microorganisms utilizing hydrocarbons as carbon and/or energy sources may be more ubiquitously present in the hadal trenches than hitherto recognized (Liu et al., 2019).

### 4.1. The non-obligate alkane-degrading nature of *Venatorbacter*

To date, four types of enzymes in the terminal oxidation of aliphatic hydrocarbons have been found in *Alcanivorax*, including *alkB* in C_5 − 18_ alkane oxidation (Liu et al., 2011; Wang and Shao, 2013), the soluble cytochromes P450 (CYP153) in C_8 − 16_ oxidation (Liu et al., 2011), flavin-binding monooxygenase *almA* in C_22 − 36_ oxidation (Liu et al., 2011), and flavin mononucleotide-binding monooxygenase *ladA* in C_15 − 36_ oxidation (Li et al., 2008). Many hydrocarbonoclastic species employ multiple *n-*alkane oxidases to utilize different alkanes, e.g., *Alcanivorax borkumensis* SK2 possesses two *alkB*, three *cyp153* (P450), and one *almA* genes (Schneiker et al., 2006).

In this study, we only identified *alkB* genes in the *Venatorbacter* genomes, and the long alkane transporters reported in *T. oleivorans* MIL-1^T^ (Gregson et al., 2018) were not identified in *Venatorbacter*. These lines of evidence indicate that *Venatorbacter* has a narrowed *n*-alkane utilization capacity and could be a non-obligate *n-*alkane degrader. Such non-obligate characteristics were also reported in the *Ketobacter* bin 12 (“*Alcanivorax* bin 12” in the original article), which was the most abundant microbe in near-bottom water of the Mariana Trench (10,500 m), and potentially could utilize *n*-alkanes, carbohydrates, amino acids, and steroids (Liu et al., 2019).

It was reported that increased HHP selectively inhibited obligate hydrocarbon-degraders and downregulated the expression of β-oxidation-related proteins (i.e., the main hydrocarbon-degradation pathway), resulting in low cell growth and CO_2_ production (Scoma et al., 2019). Metabolically, HHP induces the accumulation of citrate and dihydroxyacetone, suggesting reduced rates of aerobic oxidation of fatty acids in the TCA cycle at HHP (Scoma et al., 2019). Therefore, the utilization of other carbon sources not only alleviates the accumulation of β-oxidation products at HHP but also increases the availability of nutrients, which might especially benefit the survival of Clade VI species of *Venatorbacter*.

### 4.2. The remarkable capabilities of degrading extracellular macromolecules by *Venatorbacter*

To understand the most predominant ecological function of *Venatorbacter*, we studied the metatranscriptomic datasets from the aforementioned Von Damm hydrothermal vent plumes where *Venatorbacter* species were highly enriched (SAMN03609707 from 2,041 m and SAMN03609696 from 2,238 m, respectively). There were 26.55 and 4.5% of non-ribosomal RNA metatranscriptomic reads aligned to the corresponding MAGs (SRR2046235_bin1 and SRR2046236_bin1), respectively. This result suggests that *Venatorbacter* species were not only abundant but also greatly active in the deep-sea hydrothermal vents.

For the sample SAMN03609707, 21.86% of the 4,054 protein-coding genes of the *Venatorbacter* MAG showed more than the average transcriptional depth (73.5×). As for metabolisms, although the *alkB* genes were identified in the MAG SRR2046235_bin1, their expression was not detected *in situ* (<2×), suggesting that *Venatorbacter* species might not be a main *n-*alkane degrader in the Von Damm hydrothermal vents. However, the transcripts of carbohydrate-utilizing enzymes were identified, including D-glycerate kinase (EC: 2.7.1.31), fructose kinase (EC: 2.7.1.4), and beta-N-acetylhexosaminidase (EC: 3.2.1.52). In addition, the transcripts of ABC transporters of phospholipid (*mlaABCDE*), nucleoside (*bmpA* and *nupABC*), betaine/proline (*proXWV*), lysine/arginine/ornithine/histidine/octopine (*pa5152–5155*), and oligopeptide (*oppABCDEF*) were also identified. It suggested that *Venatorbacter* species have the capability of utilizing a wide variety of non-alkane organic substrates in deep-sea hydrothermal vents. More importantly, the type II secretion system (T2SS) is a multi-protein complex used by many bacteria to move substrates across their cell membrane, which spans the bacterial cell envelope and extrudes substrates through an outer membrane secretin channel using a pseudopilus (Naskar et al., 2021). The *mshA* and *mshI* involved in T2SS showed expression of >1,000×, and *secA* and *secY* involved in preprotein translocation exhibited expression of >700×, suggesting that protein export of *Venatorbacter* species was highly active in deep-sea hydrothermal vents (Supplementary Table S9). Accordingly, signal peptide-fused M6 family metalloprotease, peptidoglycan DD-metalloendopeptidase, phospholipase, and endonuclease were robustly expressed (>200×) in this MAG (SRR2046235_bin1). In particular, such extracellular M6 metalloprotease showed an expression of 679×, which is higher than most ribosomal proteins (Supplementary Table S9). Our experiments further confirmed that both strain C2-1 and IMCC1826 could grow with peptone as the sole carbon source (Supplementary Figure S6). These lines of evidence illustrated that *Venatorbacter* species may have a significant contribution to secondary productivity by degrading various kinds of extracellular macromolecules.

In addition, most of those genes involved in the protein secretion system and extracellular enzymes are conserved in the streamlined genome of *V. cucullus* ASxL5^T^ (with merely ~2/3 genome size of strain C2-1), suggesting that strain ASxL5^T^ specialized to be a bacterial predator *via* a massive gene loss. Considering clade VI and clade VII *Venatorbacter* species are closely related in phylogeny, strain C2-1 with the first complete genome of putatively non-predative *Venatorbacter* can be used in a comparative study with predative strains ASx5O and ASxL5 to uncover the genome reduction from a free-living ancestor to a predator in future.

## 5. Conclusion

In this study, a novel piezotolerant bacterial strain C2-1 was isolated from the deep-sea sediment of the Mariana Trench. The phylogenetic analysis showed that strains C2-1, TMPB967, ST750PaO-4, IMCC1826, TTBP476, and 41 MAGs should be classified into the genus *Venatorbacter*. Large-scale metagenomic binning and 16S rRNA gene search revealed that the genus *Venatorbacter* has a wide environmental distribution, including seawater, marine sediments, hydrothermal vent plumes, Antarctic ice, groundwater, and marine sponges. *Venatorbacter* is a non-obligate *n-*alkane degrader that can utilize, at a minimum, C_16 − 18_ alkanes, as well as carbohydrates, amino acids, peptides, and phospholipids. The identification of denitrifying genes suggested a genus-specific ecological function that allowed *Venatorbacter* to be active in anoxic environments. The robustly expressed genes in T2SS and extracellular enzymes (e.g., peptidases, phospholipase, and endonuclease) in *Venatorbacter* MAGs of deep-sea hydrothermal vents suggested the high capability of *Venatorbacter* species in degrading extracellular macromolecules. Wide environmental distribution and metabolic plasticity provide new insights into the contribution of *Venatorbacter* to biogeochemical cycling and secondary productivity in various environments.

## Data availability statement

The datasets presented in this study can be found in online repositories. The names of the repository/repositories and accession number(s) can be found below: eLMSG (an eLibrary of Microbial Systematics and Genomics, https://www.biosino.org/elmsg/index) under accession numbers LMSG_G000013420.1-LMSG_G000013457.1.

## Author contributions

JF, YW, and JW: conceptualization. YZ and YL: experiment. JW: data analysis. YW: resources. JW and YZ: writing the original draft preparation. JW, ZX, YL, JF, YW, JC, HZ, JL, TB, CS, and BL: writing, reviewing, and editing. JF: project administration. All authors contributed to the article and approved the submitted version.
