# Supplementary material for: The phylogeny and metabolic potentials of an n-alkane-degrading Venatorbacter bacterium isolated from deep-sea sediment of the Mariana Trench
Source: Front Microbiol. 2023 Mar 22;14:1108651. doi: 10.3389/fmicb.2023.1108651 (PMC10073702; doi:10.3389/fmicb.2023.1108651)
Supplement: Supplementary file 2 [file Data_Sheet_1.docx]

**Supplementary Table S1**. Cellular fatty acid composition (%) of strain C2-1 and the four closely related species type stains.

**Supplementary Table S2**. The 16S identities between strain C2-1 and related strains. The hits with <95% identities were ignored. For the genomes with heterogenous 16S rDNA sequences, only the top hits were shown.

**Supplementary Table S3**. The GC contents of the *Venatorbacter* and *Thalassolituus* species.

**Supplementary Table S4**. The average nucleotide identity (ANI) values among the *Venatorbacter* and *Thalassolituus* species.

**Supplementary Table S5**. The DNA-DNA hybridization (DDH) values among the *Venatorbacter* and *Thalassolituus* species.

**Supplementary Table S6**. The sampling information of *Venatorbacter* species.

**Supplementary Table S7**. The characteristics of C2-1, “*Thalassolituus*” *marinus* IMCC1826, *Thalassolituus oleivorans* DSM 14913^T^, *Oleibacter marinus* DSM 24913^T^, and *Oceanobacter* kriegii IFO 15467^T^, which were tested by Biolog GEN III and API 20NE.

**Supplementary Table S8**. The genes of C2-1 involved in low temperature and high hydrostatic pressure adaptation.

**Supplementary Table S9**. Gene expression of C2-1-like species in the metatranscriptional samples of Von Damm hydrothermal vent plumes, including SAMN03609707 from 2,041 m and SAMN03609696 from 2,238 m.


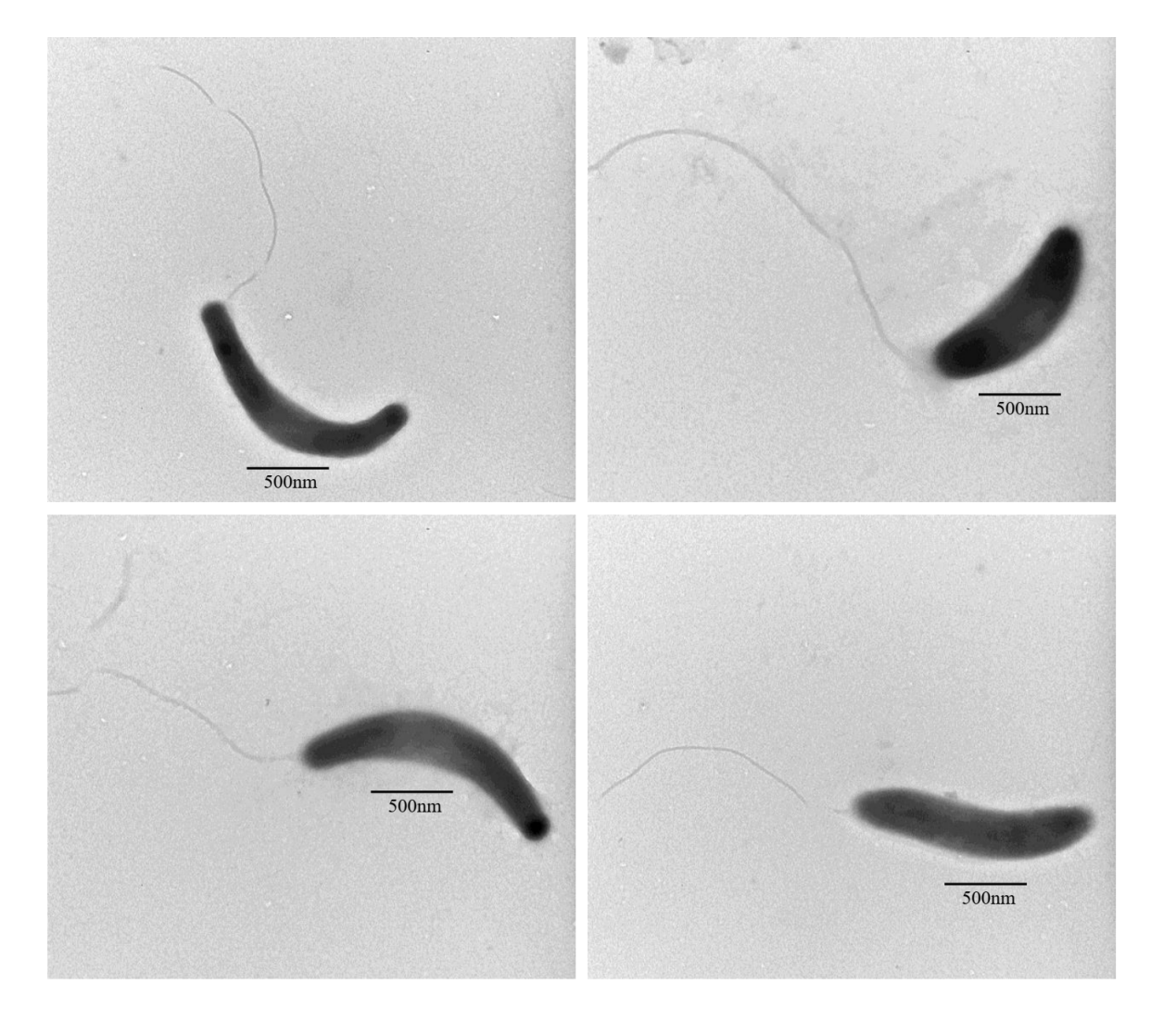
 **Supplementary Figure S1**. The transmission electron micrograph of strain C2-1


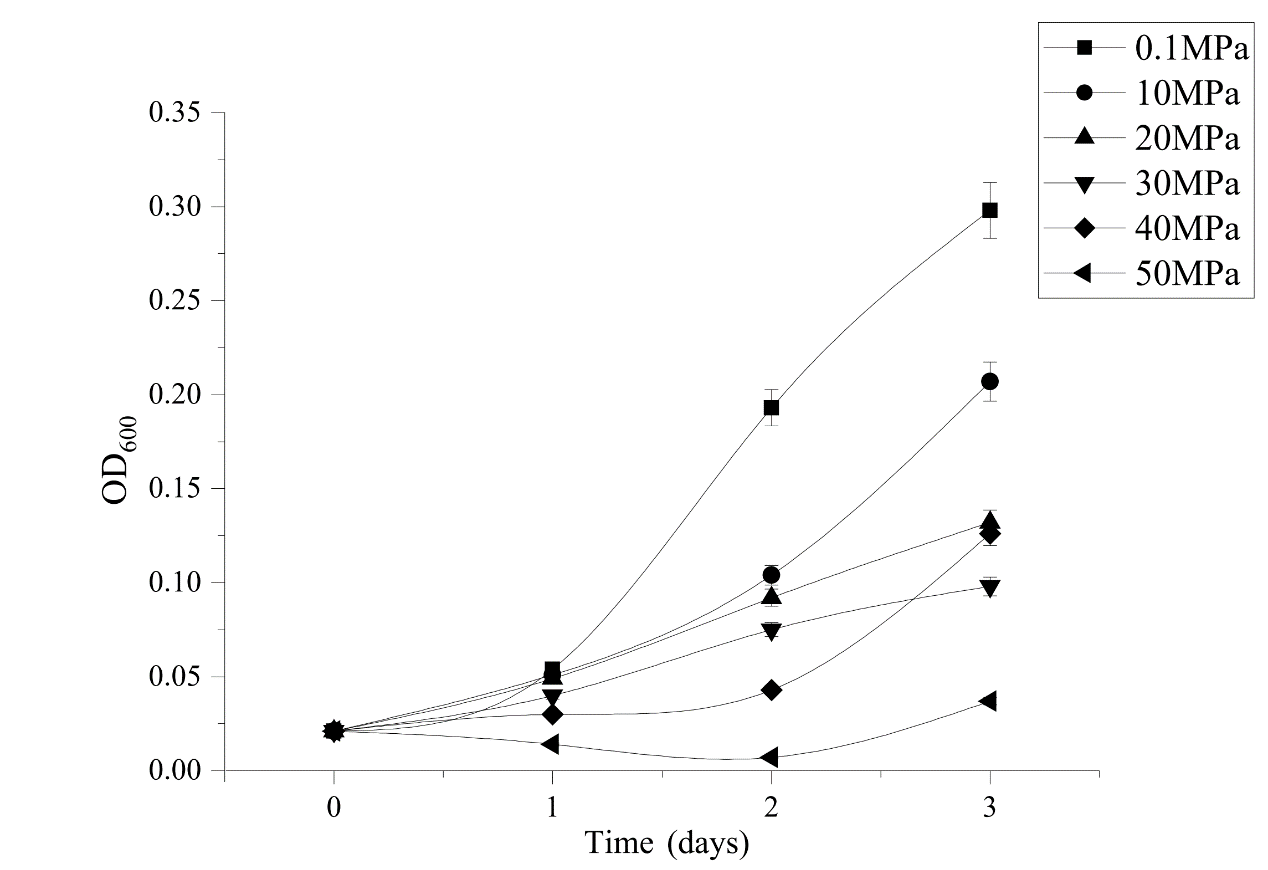
 **Supplementary Figure S2**. The growth of strain C2-1 under multiple pressures


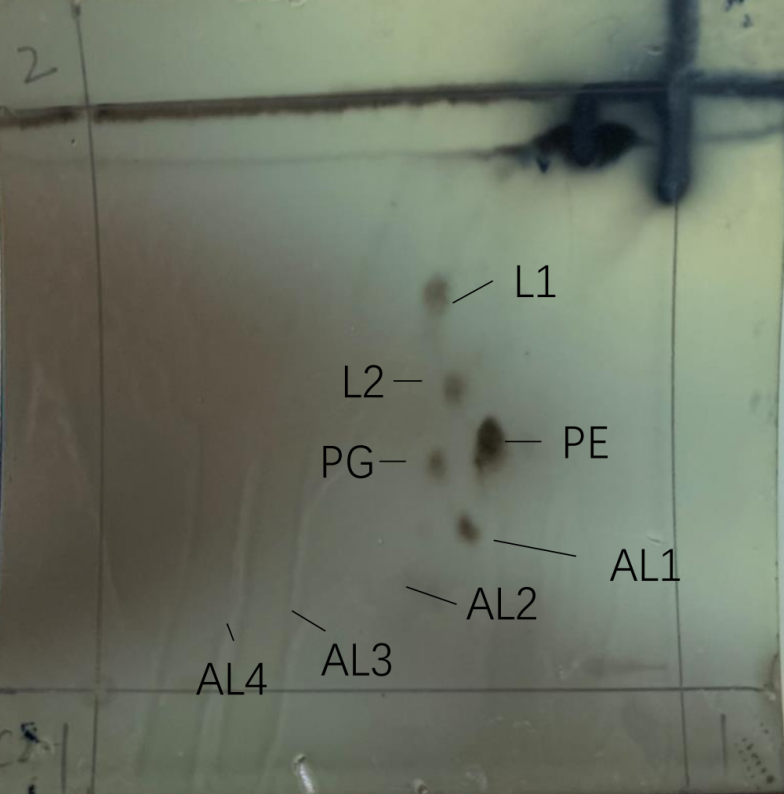


**Supplementary Figure S3**. The polar lipids of strain C2-1. PG: phosphatidylglycerol; PE: phosphatidylethanolamine; AL: aminolipid; L:  unknown Lipid


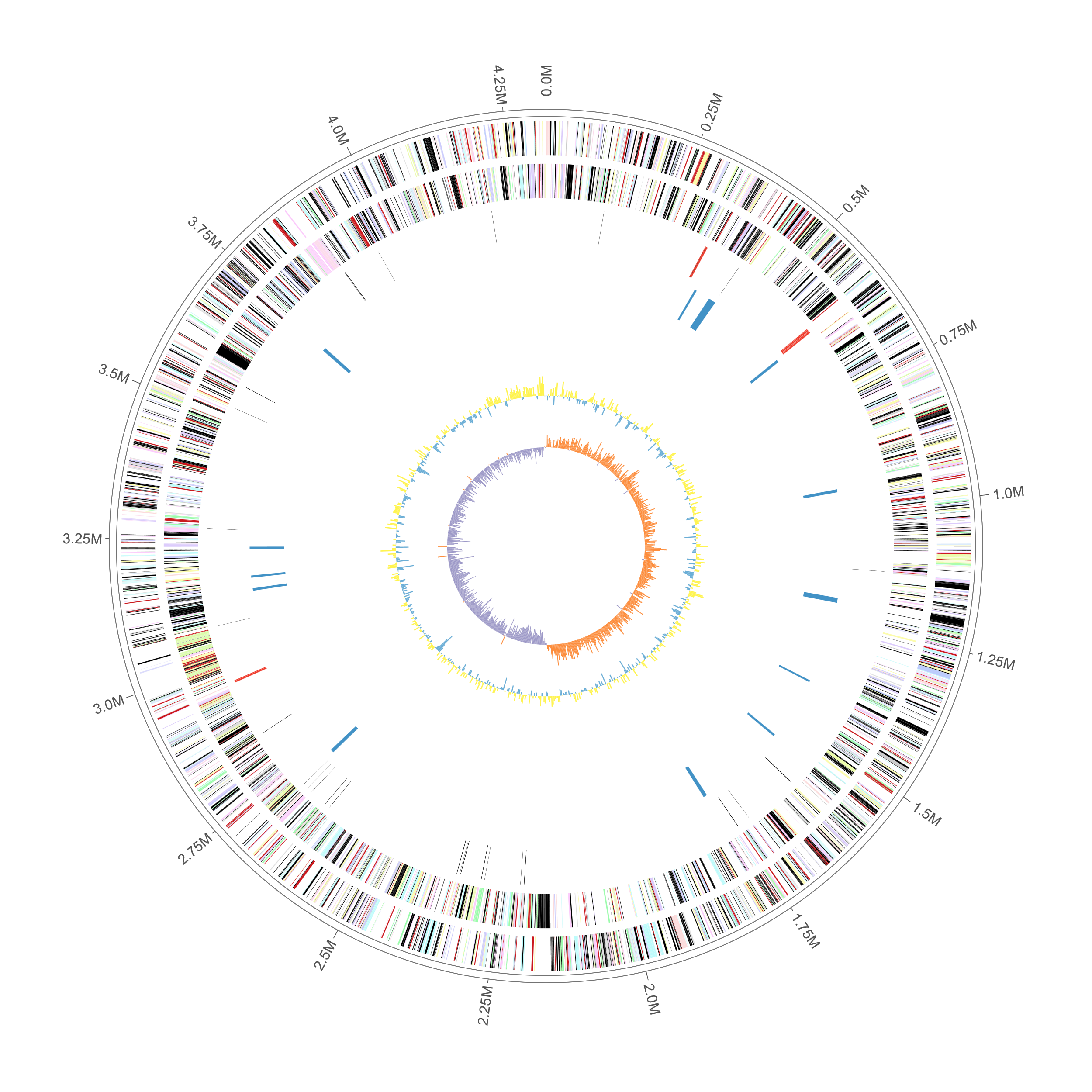
 **Supplementary Figure S4**. Graphical representation of C2-1 genome. Genes on the forward (shown in outer circle) and reverse (shown in inner circle) strands are colored according to their cluster of orthologous genes (COG) categories, RNA genes are highlighted with different colors (tRNAs black, rRNAs red), gene islands are shown in blue, GC content are shown in yellow/blue, and GC skew is shown in orange/purple.


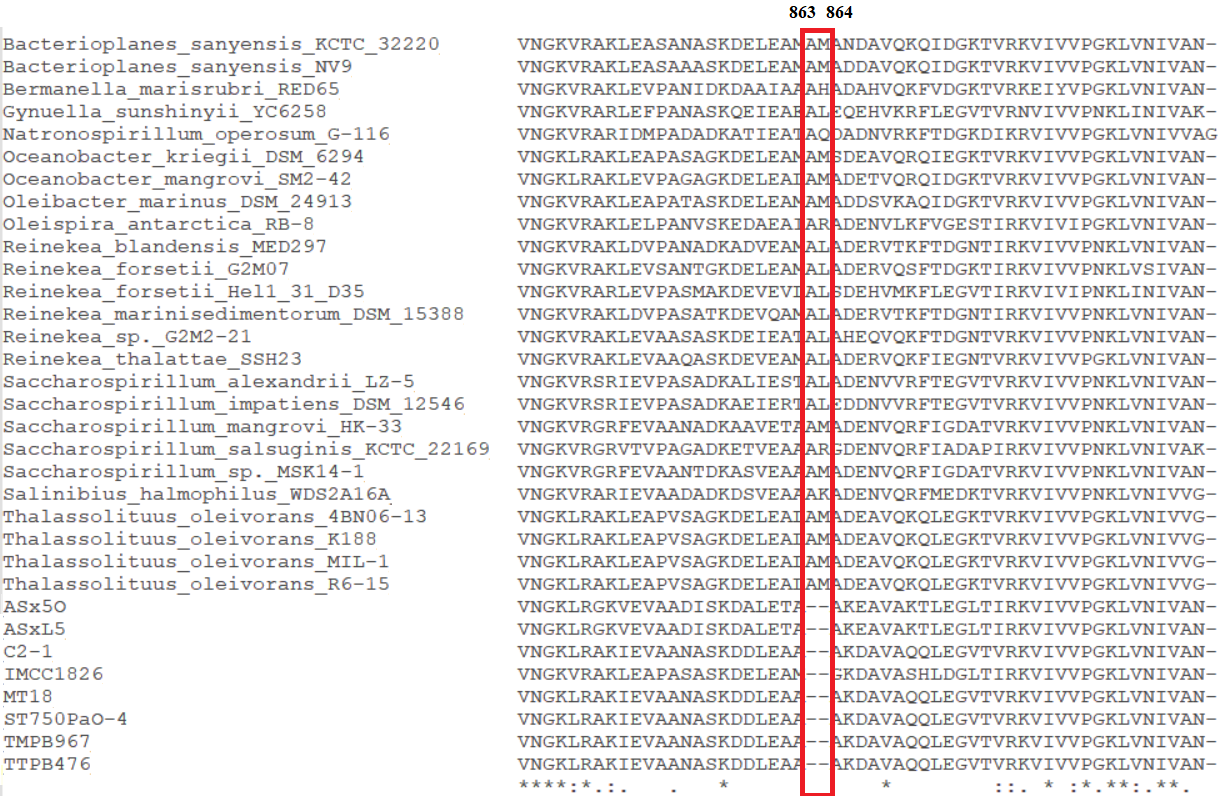
 **Supplementary Figure S5**. The genus-specific deletion in leucyl-tRNA synthetase (TIGR00396) of *Venatorbacter*. The sequences of leucyl-tRNA synthetase were identified from *Venatorbacter* and other genera belong to the family Saccharospirillaceae, including *Oleibacter*, *Thalassolituus*, *Oceanobacter*, *Bacterioplanes*, *Oleispria*, *Bermanella, Saccharospirillum*, *Reinekea*, *Gynuella*, *Natronospirillum,* and *Salinibius*.


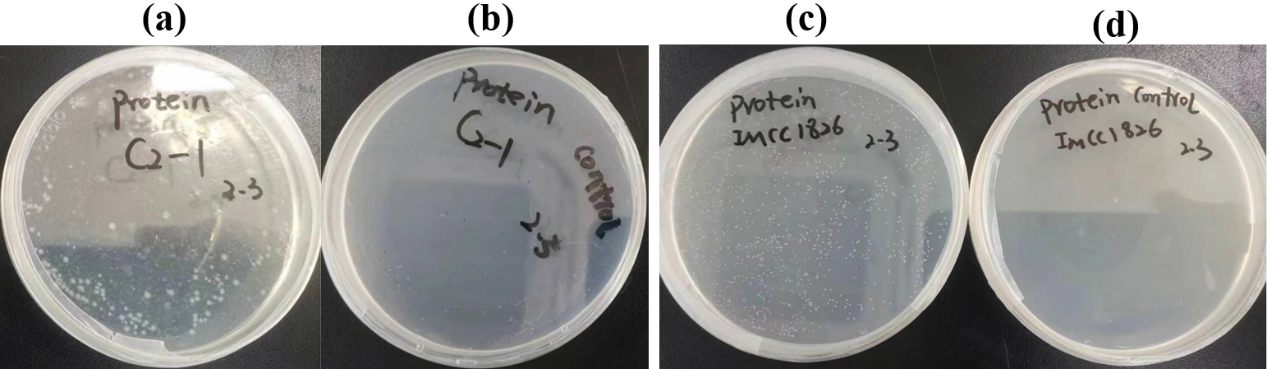


**Supplementary Figure S6**. Both strain C2-1 and IMCC1826 could grow with peptone as sole carbon source. (a) the strain C2-1 incubated with peptone; (b) the strain C2-1 incubated without peptone; (c) the strain IMCC1826 incubated with peptone; (d) the strain IMCC1826 incubated without peptone.
